# A recursive microfluidic platform to explore the emergence of chemical evolution

**DOI:** 10.3762/bjoc.13.164

**Published:** 2017-08-17

**Authors:** David Doran, Marc Rodriguez-Garcia, Rebecca Turk-MacLeod, Geoffrey J T Cooper, Leroy Cronin

**Affiliations:** 1 WestCHEM, School of Chemistry, University of Glasgow, University Avenue, Glasgow G12 8QQ, UK

**Keywords:** artificial life, autocatalysis, automated platforms, chemical evolution, evolution before genes, evolution first, microfluidics

## Abstract

We propose that a chemically agnostic approach to explore the origin of life, using an automated recursive platform based on droplet microfluidics, could be used to induce artificial chemical evolution by iterations of growth, speciation, selection, and propagation. To explore this, we set about designing an open source prototype of a fully automated evolution machine, comprising seven modules. These modules are a droplet generator, droplet transfer, passive and active size sorting, splitter, incubation chamber, reservoir, and injectors, all run together via a LabVIEW^TM^ program integration system. Together we aim for the system to be used to drive cycles of droplet birth, selection, fusion, and propagation. As a proof of principle, in addition to the working individual modules, we present data showing the osmotic exchange of glycylglycine containing and pure aqueous droplets, showing that the fittest droplets exhibit higher osomolarity relative to their neighbours, and increase in size compared to their neighbours. This demonstrates the ability of our platform to explore some different physicochemical conditions, combining the efficiency and unbiased nature of automation with our ability to select droplets as functional units based on simple criteria.

## Introduction

The transition from an inanimate inorganic world, principally consisting of minerals, gases and small organic compounds, to the living world with the first life forms remains one of the greatest mysteries in science [1]. In the early 20th century, Alexander Oparin and John Haldane proposed that the first minimal living systems on Earth formed via a series of chemical steps of increasing sophistication and functionality. In subsequent decades, knowledge of the materials and environments that would have been available on the early, prebiotic Earth has expanded dramatically [1–5]. This has enabled the reduction of the potential chemical and geochemical landscape for abiogenesis from a vast parameter space, but has also led scientists to propose hypotheses on the origin of life under very constrained conditions [6].

Many heated debates in the field of prebiotic chemistry have raged over which precise historical environment(s) gave rise to the first lifeforms. However, it is unlikely that this question can ever be answered with reasonable certainty [7]. Therefore, the puzzle most ripe for scientific inquiry is not how did life first arise, but what kind of processes can facilitate the origin of life? Identification of processes that produce complex, autocatalytic chemical networks [8] from simple inputs via gradual, step-wise complexification could go some way towards answering the latter question. This approach engenders a “chemically agnostic” perspective, in which strict adherence to the chemical repertoire found in currently extant biochemistry is not required [9]. Indeed, the simplest biological units can be considered as nothing more than complex autocatalytic networks that reproduce, with more or less the same stoichiometry, all functionally active components of their heterogeneous chemical mixtures. Such systems could easily exist outside the boundaries of known biology, and perhaps may not even require a template-driven genetic polymer to reproduce [10].

However, irrespective of their chemistry, it is likely that any artificial or alternative life-forms would need at least the following attributes:

i) Compartmentalisation: a means of discretising individual living units and enabling controlled selective exchange between these units and their external environment.

ii) Metabolism: chemical reaction networks that extract energy from the environment in a useable form.

iii) Heritance: reliable transmission of functional information from one generation to the next.

iv) Evolution: a means of undergoing an evolutionary selection process, driven by errors or variations in the heritance process.

Attempts to facilitate the emergence of adaptive evolution in artificial systems have been fraught with difficulties. A lack of clear, tangible criteria for identifying this process when it occurs has hindered efforts to create artificial life. The hallmark of evolution is adaptation in response to selection pressure and environmental change. Evolutionary biologists often track this process using biochemical signatures such as genome sequence. However, this would be difficult in artificial or otherwise alternative life, especially if there is no conventional, template-directed genetic system. Thus, the first step is to establish a suitable metric for identifying and measuring their capacity for evolution.

We propose that, for any given population of discrete living or proto-living units, the average fitness (*w*_i_) of the population will be evaluated as a function of time, environmental change (Δ*e*_i_) and population size. Fitness will be determined and thresholded by intensity of an observable, quantitative trait (*z*). Only those units with a fitness exceeding a pre-determined threshold (*f*) will be permitted to reproduce and pass on information to the next generation. Repeating this process in an iterative manner, allowing only the fittest members of each generation to affect the chemical composition of subsequent generations, will lead to adaptive evolution.

[1]
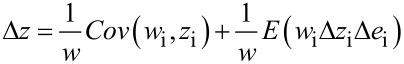


Where *Cov* = covariance and *E* = sample mean.

Equation 1 is a modified Price equation [11] with the change in the environment Δ*e*_i_ factored into the second term which is nominally *E*(*w*_i_Δ*z*_i_). Evolution in any given system will be confirmed via a successive change in Δ*z* over time.

**Figure 1:**
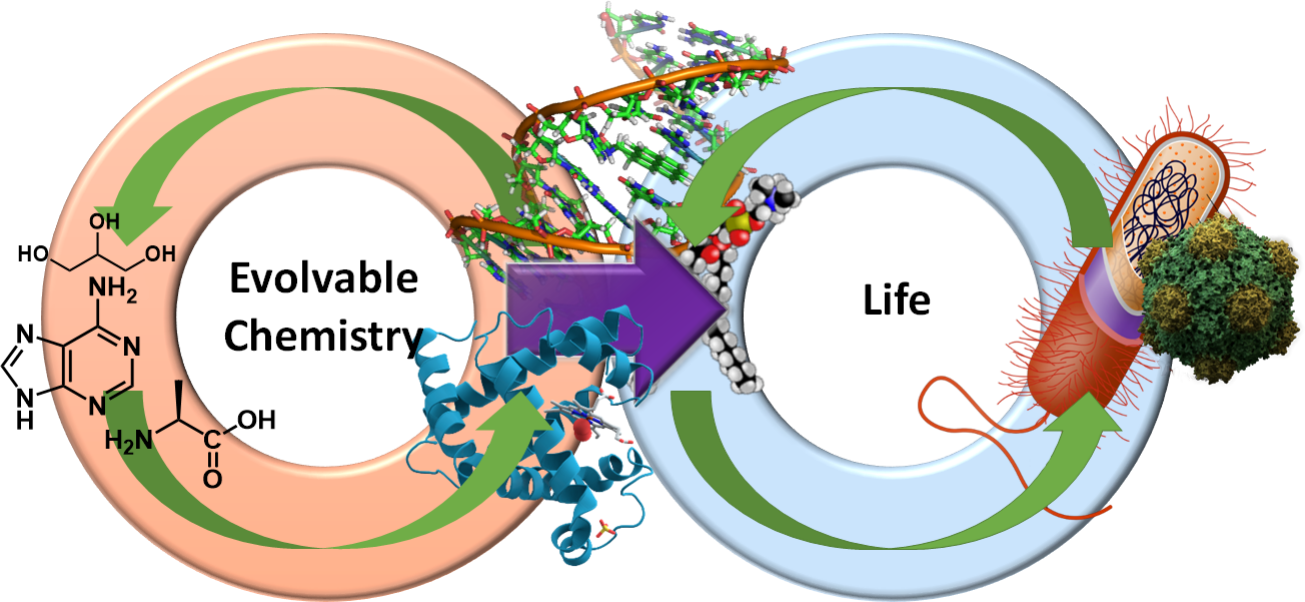
Evolution of life from non-living, complex chemistry via chemical evolution of complex chemical composites towards increasing complexity. A transition to biological evolution occurs when composites become sufficiently complex to transition from chemical to biological units. Green arrows indicate continuous adaptation and complexification under selection pressure; the purple arrow indicates the transition from evolving chemical composites to evolving living units after exceeding a complexity threshold.

Evolvability is a pre-requisite for life, but it is not sufficient for a system to be deemed living or life-like. Therefore, our group is also developing a metric for evaluating the complexity of chemical species produced in artificially evolving systems. This complexity measurement will be thresholded using existing biological systems and by comparison with the starting inputs into our evolutionary platform. An artificial living system would be capable not only of evolution, but also of producing species with a greater complexity than would be expected to arise from any non-biological system [12]. Thus, the transition from an evolving but non-living chemical system to an evolving living system will be marked by production of species of comparable complexity to those found exclusively in biology, as depicted in Figure 1.

### Droplet compartmentalisation

**Figure 2:**
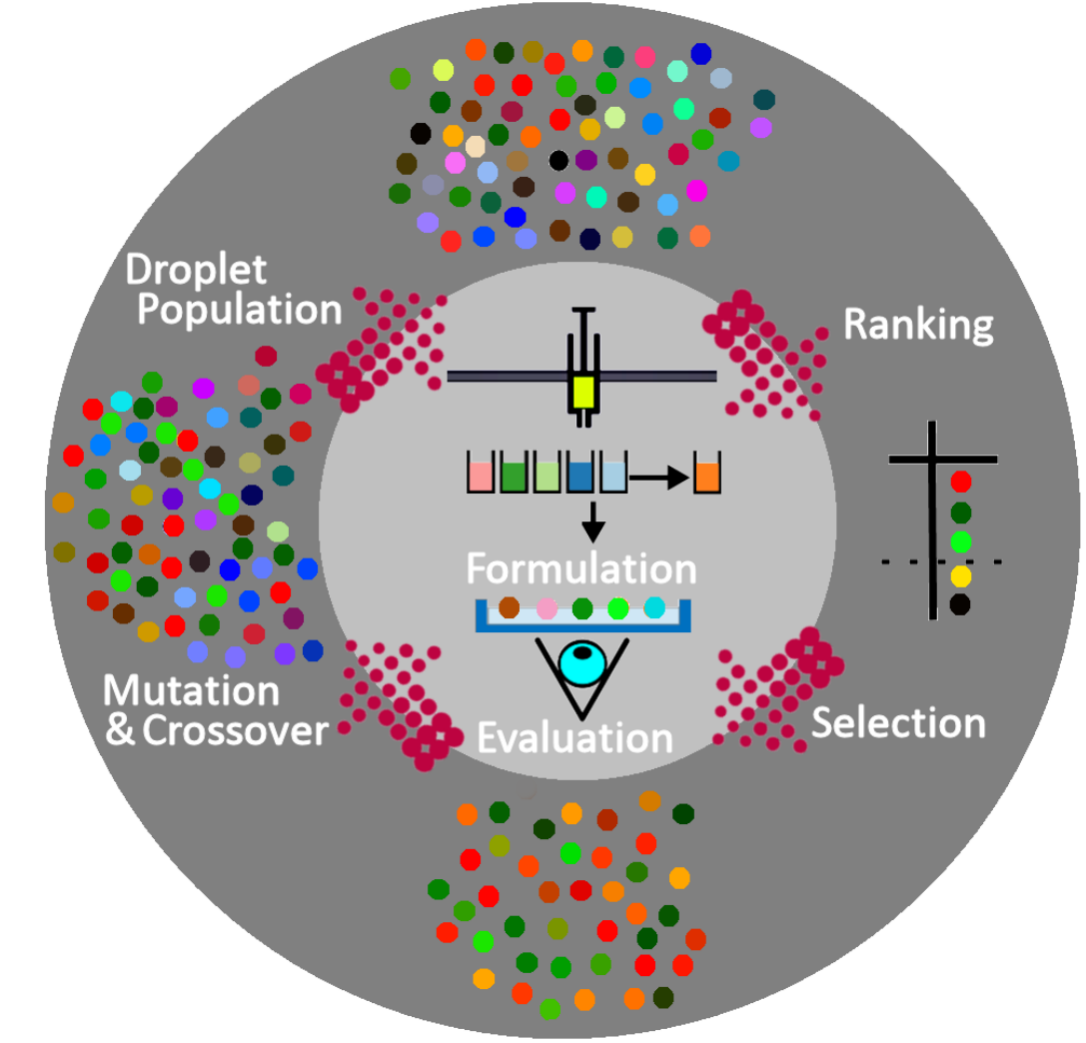
Schematic describing the evolutionary process. The inner circle represents the robotic process and outer circle represents the computational algorithm. A random selection of the droplet formulations are used as the starting ‘Droplet Population’. These droplets are generated in the ‘Formulation’ step. Droplet behaviours are then recorded using a camera, and then undergo image analysis against a user desired property (e.g., colour) in the ‘Evaluation’ step. The droplets are ranked in terms of desired property (e.g., movement, division), and the least good rejected in the ‘Ranking’ step allowing a new population to be ‘Selected’. Meanwhile the accepted formulations are used as a basis to create a new ‘Droplet Population’ after random ‘Mutation’ and ‘Crossover’. This figure was reproduced from our earlier article [13], copyright 2014 Macmillan Publishers Limited.

In our previous work, we described the assembly of a custom-made 3D printed robotic platform that uses artificial evolution to select for desired behaviours in chemical systems [13]. In this case, the macroscopic behaviour of oil droplets was studied. We used a genetic algorithm to generate a series of droplets, each with a different set of chemical compositions, which were evaluated according to various fitness functions based on observable traits, such as motility, vibration and division. The chemical mixtures that produced the fittest droplets in each generation of experiments were allowed to influence the compositions of the next generation, while the rest were discarded. As this process was repeated iteratively over successive generations, the fitness value of the population was increased (Figure 2).

Droplets provide a means of creating discrete, compartmentalised units, defining the “self” or units of evolution. These defined units can then be subject to conventional selection processes. The work described above was carried out in microlitre scale droplets. However, a few recent examples in the literature report the utilisation of pico- and nanolitre microfluidic water-in-oil droplets and liposomes as artificial cell analogues [14–15]. Aqueous, single emulsion microdroplets can be produced at kilohertz frequencies, and provide compartmentalisation on a similar length scale to biological cells. Soft interface interactions at liquid–liquid boundaries in microdroplets can also have a catalytic effect via the adsorption of otherwise unstable molecules [16], similar to catalysis reported at liquid–mineral interfaces [17].

### Microfluidic platform for artificial evolution in droplets

Here, we propose a system for facilitating chemical evolution in populations of co-incubating aqueous, single emulsion microfluidic droplets.

**Figure 3:**
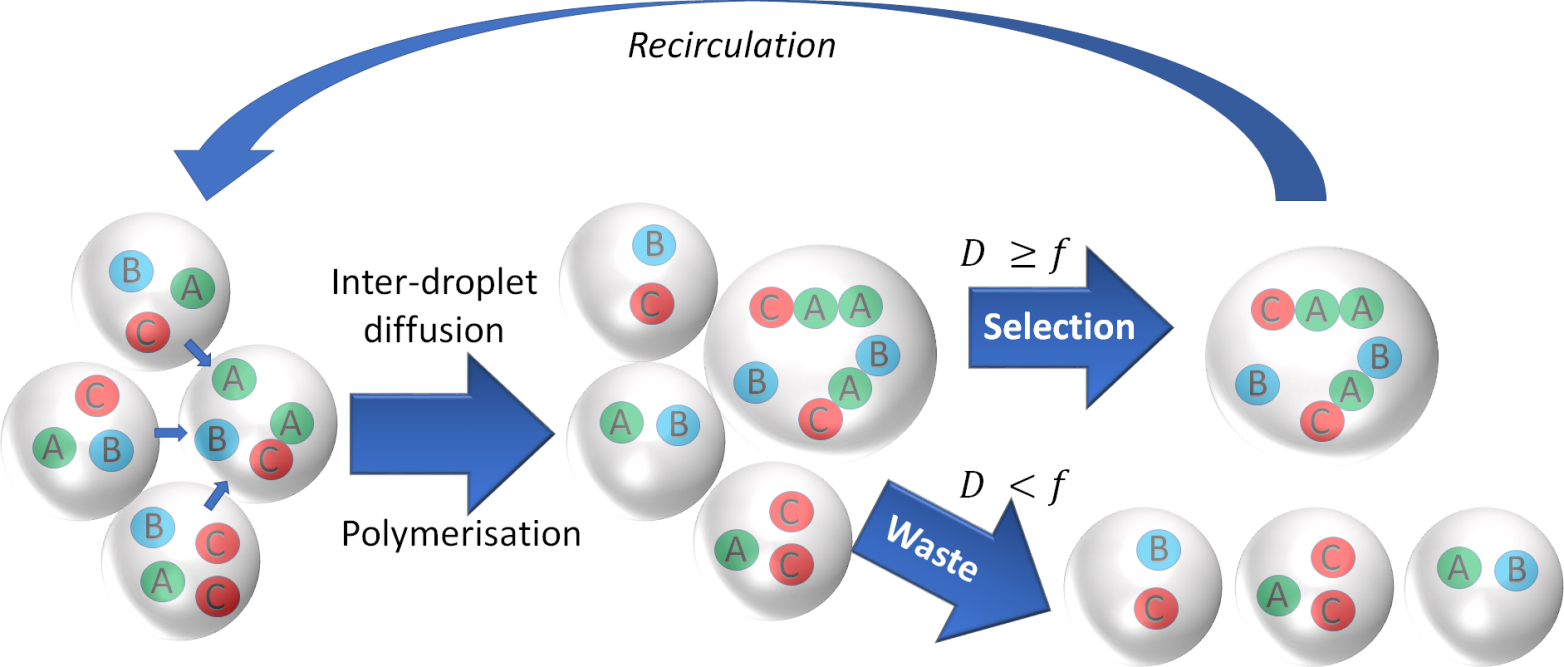
Recursive size-based selection and recirculation of droplets. Monodisperse droplets loaded with complex autocatalytic chemical networks are incubated in the microfluidic device. Those droplets which facilitate the fastest production of high molecular weight polymers from simple precursors exhibit an increase in osmolarity and subsequently grow in size at the expense of neighbouring droplets. Size sorting is then used to select for droplets with a diameter (*D*) above a size threshold for droplets to be used in the next generation (*f*). Those droplets are recirculated into the next generation, replenished with fresh feedstock, and the process is repeated in an iterative fashion.

Each microdroplet can be considered an autonomous microreactor, loaded with a self-propagating chemical reaction network. However, it has been observed, both in our own work and in the literature that limited exchange of material can occur between neighbouring water-in-oil microdroplets (see Figure 3). The rate of diffusion of molecules between microdroplets is inversely proportional to their molecular weight, with the result that microdroplets containing higher molecular weight species exhibit greater osmotic pressure, and thus physically grow in size at the expense of their neighbours via osmotic effects [18–20]. This is particularly the case when microdroplets contain reactions that convert relatively simple, low molecular weight starting materials into larger, more complex products. Such a set-up is amenable to inducing competition and evolutionary selection pressure within populations of microdroplets, using physical droplet growth as a fitness metric. The quicker droplets can produce larger, more complex products, the more likely they are to grow. Size sorting can then be applied to select for the fittest, fastest growing droplets and ensure only these droplets are recirculated in the next iteration of reaction and selection.

## Results and Discussion

**Figure 4:**
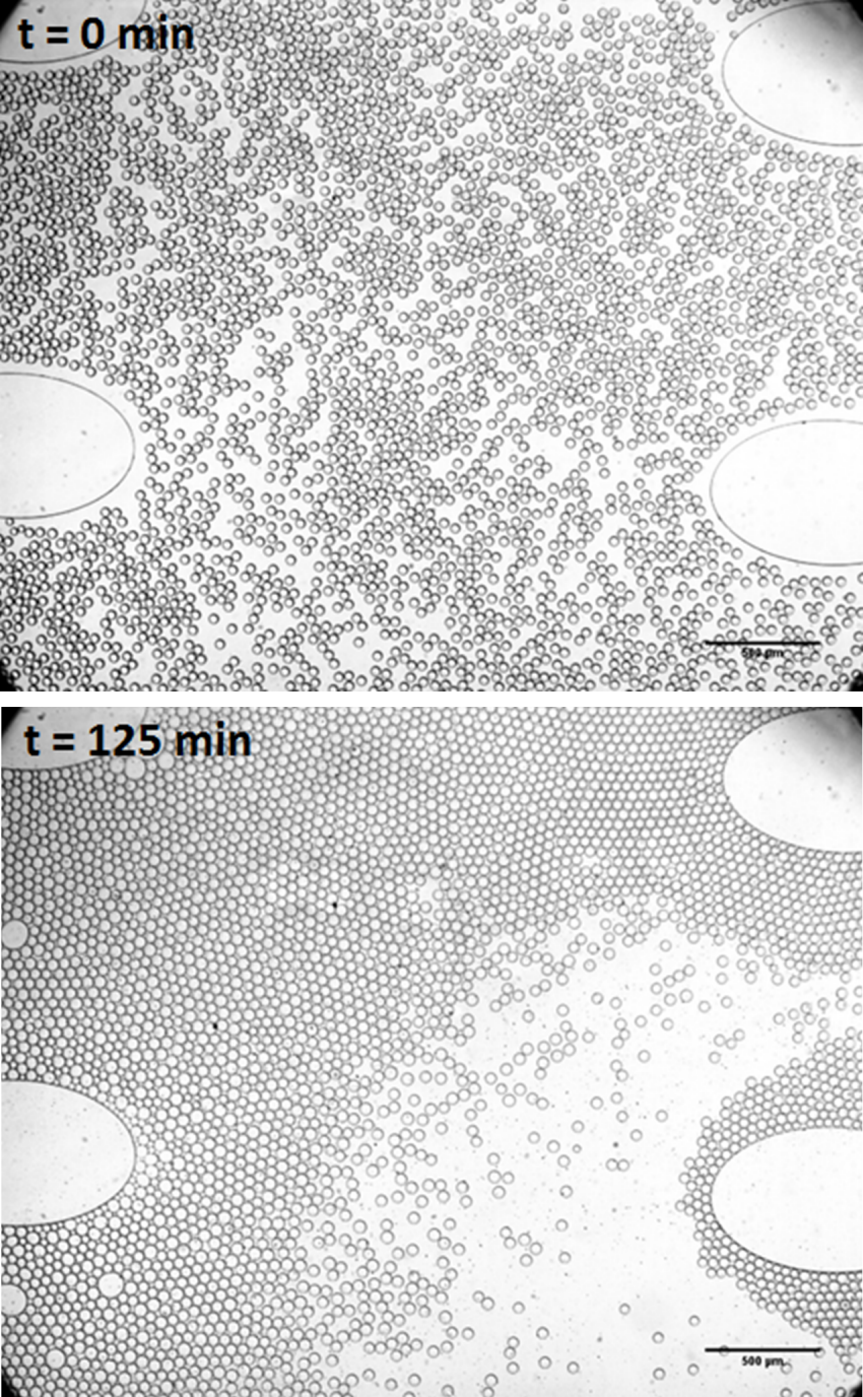
Osmotic exchange and coarsening of co-incubating aqueous microdroplets. 50 mM glycylglycine and pure H_2_O droplets were co-incubated in the same chamber. Upper panel: mixed but monodisperse droplet population at *t* = 0 min; lower panel: *t* = 125 min.

**Figure 5:**
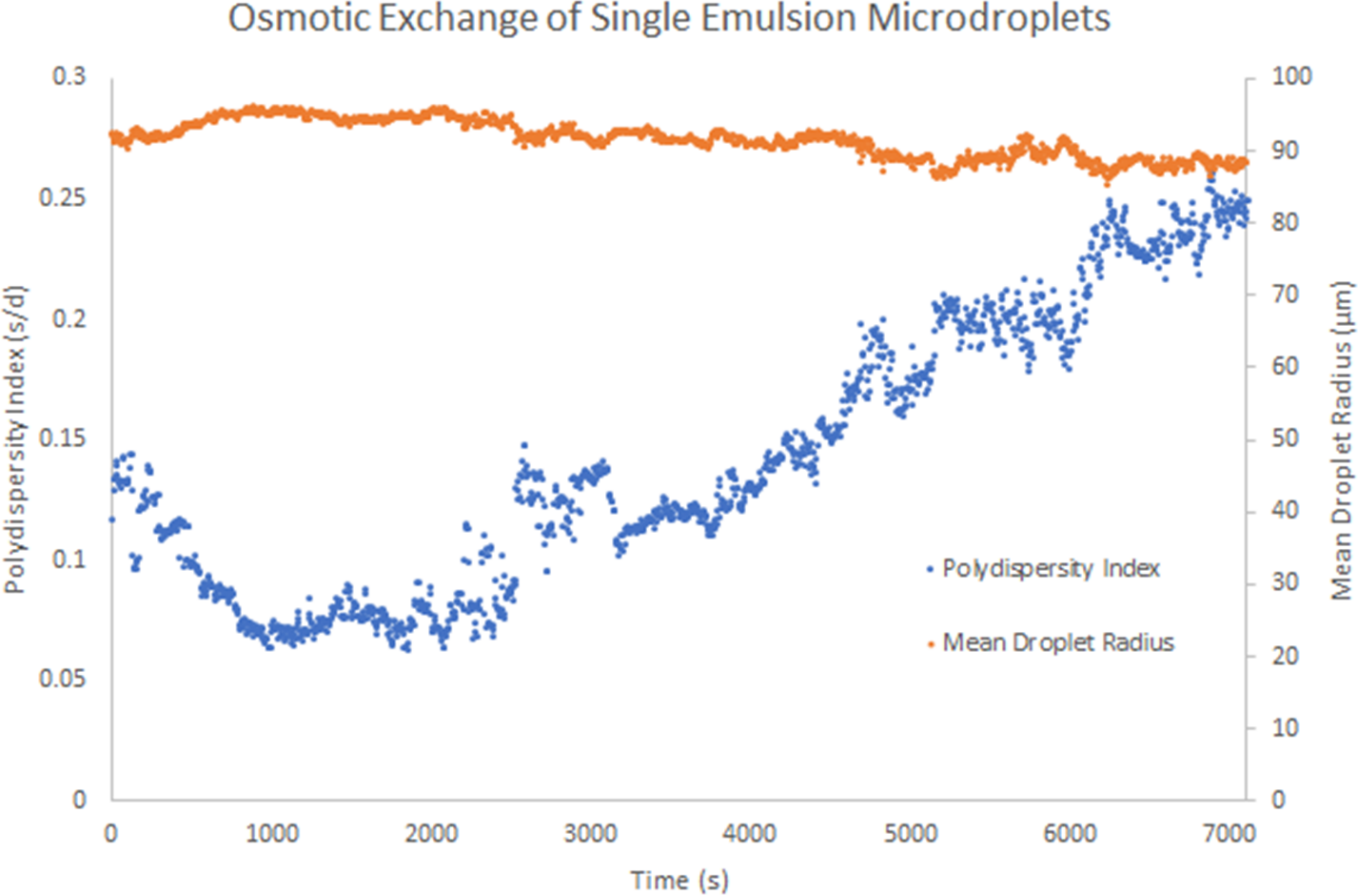
Real-time, LabVIEW^TM^ tracking of osmosis-driven coarsening of 50 mM glycylglycine and pure water droplets. Increase in droplet polydispersity is monitored using LabVIEW^TM^ image analysis. *s* = standard deviation of droplet radius, *d* = mean radius.

To test the ability of aqueous droplets to grow at the expense of each other we undertook some experiments to explore osmotic exchange between microdroplets. A mixed but monodisperse population of 50 mM glycylglycine droplets and pure water droplets was used as a model for this process. Due to their greater osmotic pressure, the glycylglycine droplets grew at the expense of the water only droplets (Figure 4 and Figure 5). This effect was not observed for unmixed droplet populations containing only glycylglycine or pure water. Using LabVIEW^TM^ image analysis, the osmotic exchange process can be tracked in real time by measuring average droplet size and polydispersity (Figure 5).

Various microdroplet size sorting techniques [21–22] can be used to enforce a positive selection pressure for increase in droplet size. By doing this iteratively, over multiple generations and ensuring a continuous (but not unlimited) supply of fresh feed-stocks, it will be possible to observe the emergence of adaptive evolution. Differential fitness can then be induced in droplets when they are forced to compete for the same feedstocks [23]. Successive increases in the rate of droplet growth could be indicative of evolutionary processes in response to the continuous selection pressure. In parallel, the chemical composition of microdroplets will be analysed after each iteration.

**Figure 6:**
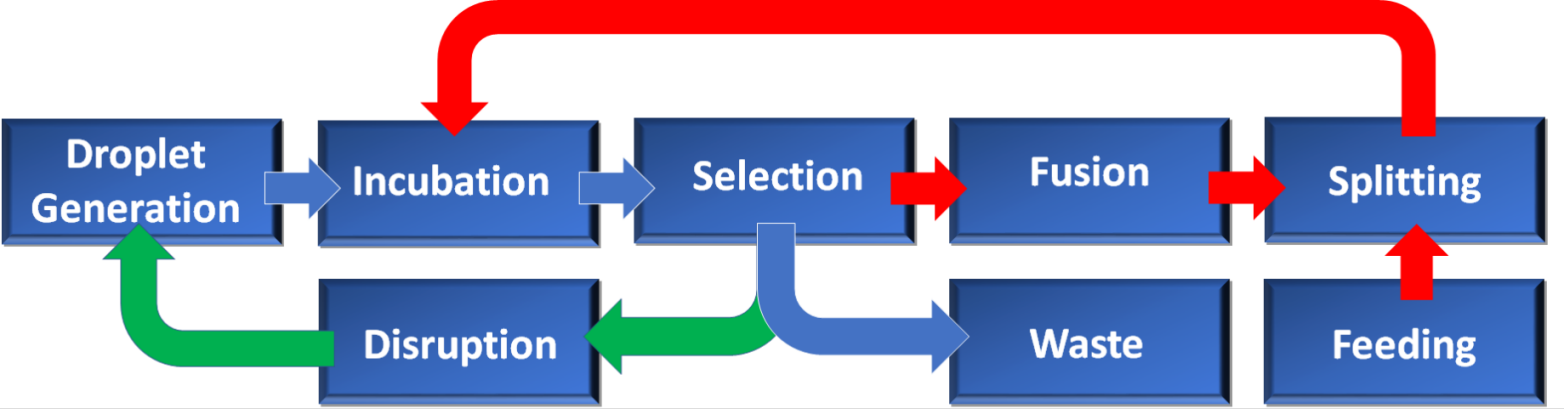
Process of the automated microfluidic platform, in which recursive evolution is applied at both individual droplet and sub-population level. Blue arrows indicate processes common to all devices; green arrows indicate processes unique to sub-population selection; red arrows indicate processes unique to individual droplet selection.

In principle, this device should be able to carry out multiple cycles of automated droplet generation, manipulation and selection, as shown in the process diagram in Figure 6. Passive and active size sorting methods will be used for selection of droplets in sub-populations and as individuals, respectively. For active sorting, real-time image processing will be used to screen individual droplets as they pass through a microfluidic channel. If the droplets exceed a pre-defined size threshold for fitness, an air-actuated polydimethylsiloxane (PDMS) valve will be activated and the droplets will be isolated and put through a new round of growth and selection. Passive sorting techniques (such as pinched flow fractionation) [22] have been used to sort droplets into groups (or sub-populations). This process can also be monitored in real-time, but this is not a requirement for the droplet sorting and selection to proceed. Also, unlike active sorting, passive sorting is not reliant upon automation, and is therefore technically less complex. In both systems, droplets below a critical size threshold for fitness are discarded.

**Figure 7:**
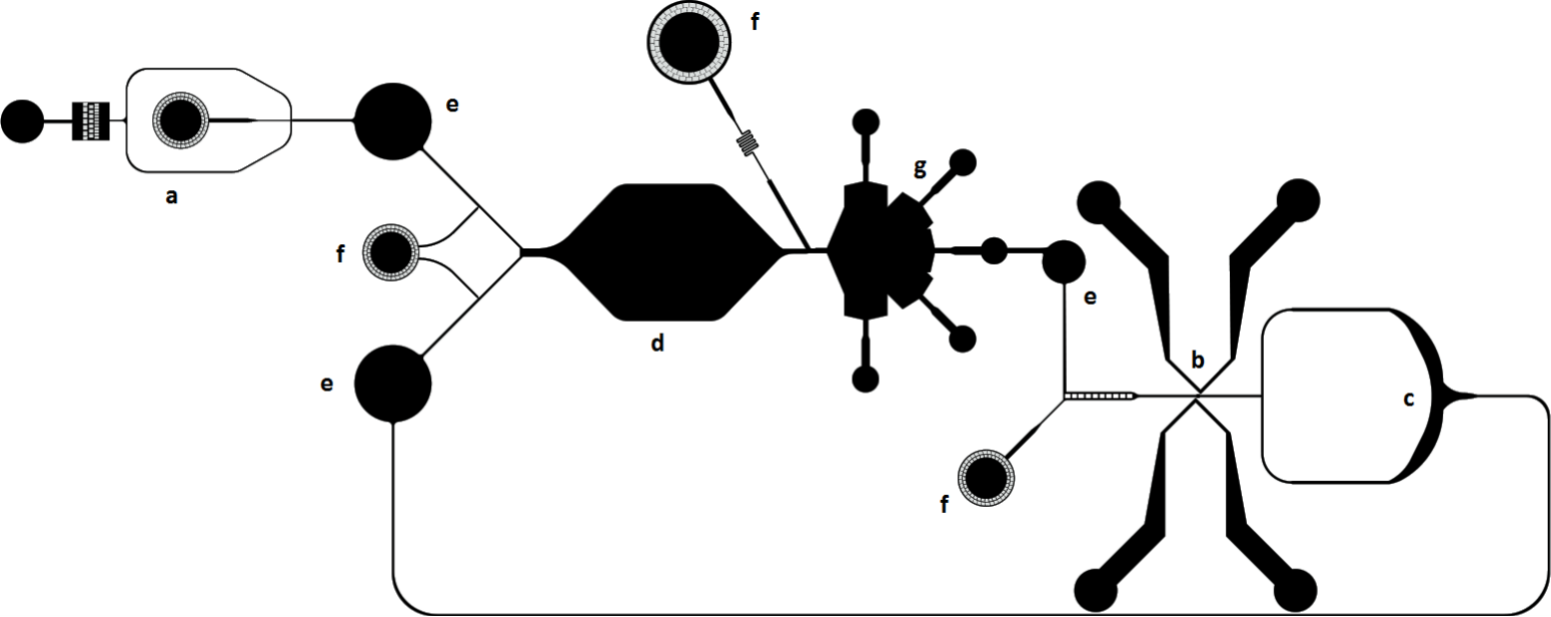
The proposed device for droplet selection and evolution. The device is comprised of the following modules: a) droplet generator; b) droplet fuser; c) droplet splitter; d) incubation chamber or delay line; e) droplet packing reservoirs; f) oil injectors; g) droplet size sorter.

Our aim is to design and fabricate a complete device containing a droplet generator, an incubation chamber, a droplet size sorter, a droplet fuser, and a droplet splitter; see Figure 7 for the device template. Microfluidic droplet generators will produce the droplet populations that will then be co-incubated in different environments (e.g., pH, salt, temperature, surface chemistry, chemical inputs). Droplets that are able to grow sufficiently will be re-circulated with fresh feedstocks for further cycles of incubation and selection, whilst droplets that get smaller will be discarded. Thus, a continuous selection pressure for droplet growth will be enforced in a recursive manner.

While operating such a device with many interconnected (but independently operating) modules can be challenging, we control timing and feedback issues using interspersed packing reservoirs (Figure 7e) and actuated mechanical valves. The packing reservoirs represent 3-dimensional structures that take advantage of the tendency of aqueous droplets to float in the surrounding fluorinated oil, and require an external outlet below the device (to allow for excess oil drainage) connected to an automated valve. This has been done successfully in our lab using syringe pumps, but could be controlled through other automated means. The addition of air pressure-actuated valves throughout the device should also help to control the timing of droplet movement, and experimentation will determine at which points in the device these valves are necessary. The incubation chamber (Figure 7d) represents a means of visualising a monolayer of droplets over time, which could be useful if we are looking to monitor the droplet coarsening process over time. However, this module could be replaced by a delay line or an off-chip incubation receptacle if the experimental parameters are not conducive to long-term on-chip incubation. Finally, successful operation of the device will depend on automated movement of the microscope stage to focus on the different modules, along with collecting visual data for the purposes of tuning rates of flow for the individual modules to carry out their functions.

**Figure 8:**
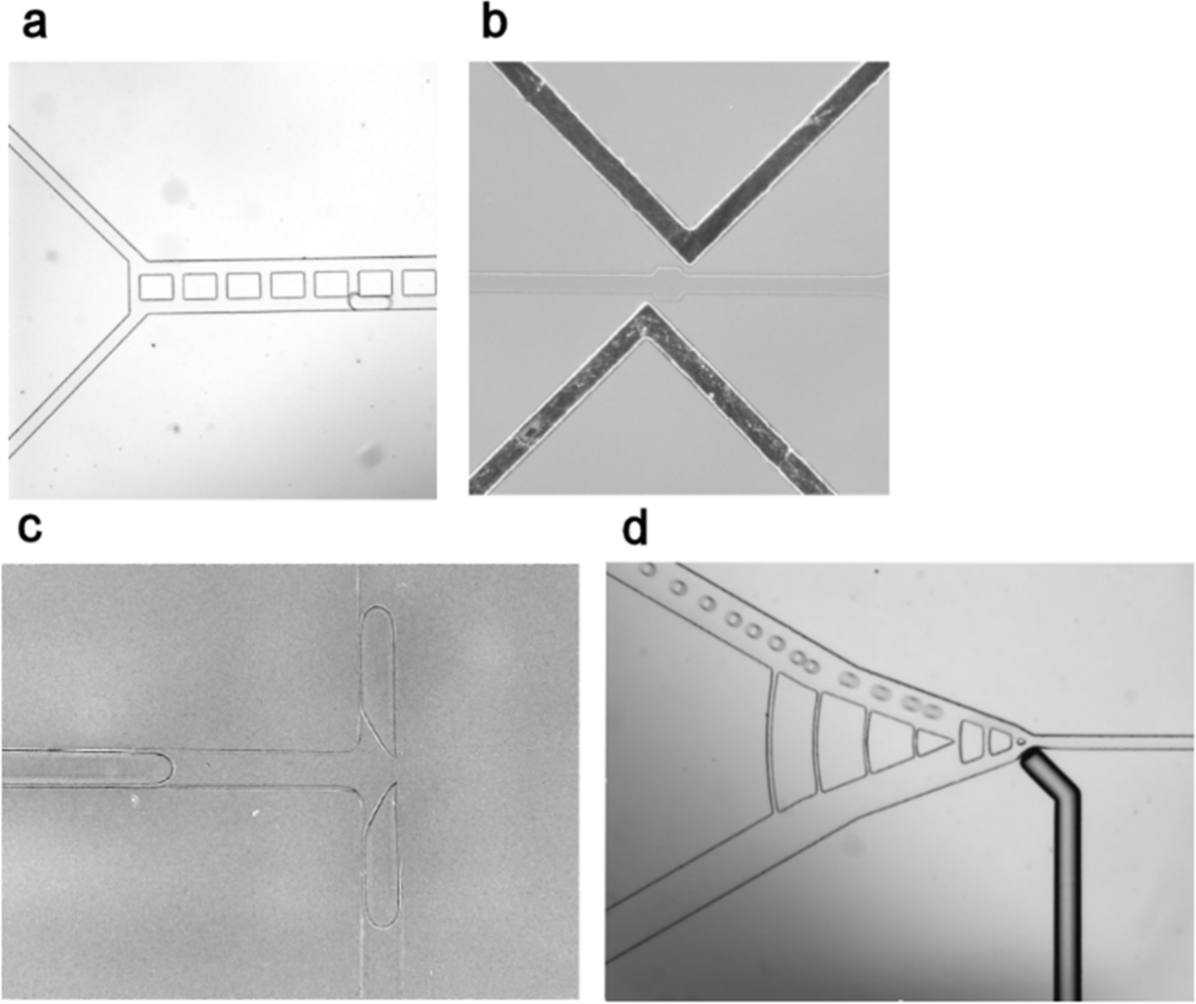
Photographic images of individual microfluidic modules, fabricated our laboratory in PDMS from standard soft lithography masters: a) droplet synchroniser for b) droplet fuser; c) droplet splitter; d) droplet sorter with air pressure-activated mechanical valve.

Also, to test if the platform is feasible, we have made several working versions of the modules (Figure 8) which include a droplet sorter. Droplet synchroniser, droplet fuser, and droplet splitter modules are required for replenishment of microdroplets with fresh feedstocks. However, in the future droplet chemistry could be adjusted so as to allow spontaneous droplet division, thus imparting a greater degree of autonomy (and thus “aliveness”) in the system.

## Conclusion

We have presented a new conceptual approach, and platform design, to search for chemical systems within an automated microfluidic platform that allows the creation of a population of individuals, the application of selection pressure, selection, combination, then splitting of the members of the population. We have produced each of the modules individually in our laboratory, but integration into a single device will be a bigger challenge. However, the exploration of osmotically driven droplet growth has been successful and this is an important step in producing populations of droplets with different chemical constituents capable of guest exchange. This will be done by recirculating droplets that meet our fitness criteria and combining them with new droplets from our variable input system. The evolutionary capacity of droplet units will be evaluated by the modified Price equation (Equation 1), with change in droplet size being equivalent to Δz. In this way, we can search for emergent physical properties of compartmentalised systems in an unbiased and fully automated manner.

We have already designed, fabricated and tested several of the individual modules in single-layer PDMS devices that comprise the platform. The chemical inputs, selection pressure, and population size will be varied as a function of cycle number. As the fitness of the population approaches a threshold we will investigate the populations for evidence of the emergence of life like properties ‘evolved’ within the device. With this approach, we suggest that such a platform may allow compartmentalised chemical units to undergo a process like evolution at the chemical level.

## Supporting Information

File 1 Additional material.
