# Supplementary material for: A recursive microfluidic platform to explore the emergence of chemical evolution
Source: Beilstein J Org Chem. 2017 Aug 17;13:1702–9. doi: 10.3762/bjoc.13.164 (PMC5564272; doi:10.3762/bjoc.13.164)
Supplement: File 1 — Additional material. [file Beilstein_J_Org_Chem-13-1702-s001.pdf]

Supporting Information

for

A recursive microfluidic platform to explore the emergence of chemical evolution

David Doran, Marc Rodriguez-Garcia, Rebecca Turk-MacLeod, Geoffrey J. T. Cooper and Leroy Cronin*

Address: WestCHEM, School of Chemistry, University of Glasgow, University Avenue, Glasgow G12 8QQ, UK

Email: Leroy Cronin - Lee.Cronin@glasgow.ac.uk

*Corresponding author

Additional material

Table of Contents

1. Device fabrication... S2

2. Microfluidic operations... S2

2.1 Droplet generation... S2

2.2 Droplet incubation and transfer... S3

2.3 Droplet size sorting... S4

2.4 Droplet splitting and fusing... S5

2.5 Osmotic exchange in droplets... S6

3. Labview integration and image analysis... S6

4. Instrumentation and materials... S10

1 Device Fabrication

Devices were fabricated using standard soft-lithographic techniques from master moulds of polymerised TMMR S2000 negative photoresist, a photoactive cross-linker, by spin-coating onto a 100 mm diameter silicon wafer. Photomasks were designed using Rhinoceros 5 vector drawing software, and printed on high-resolution acetate films purchased from Micro Lithography Services Ltd. UV lithography was used to pattern the photomask design onto silicon wafers (Figure S1). Wafers were used as a positive stamp for casting and replica moulding of polydimethylsiloxane (PDMS), which were then plasma bonded onto a glass substrate using an oxygen plasma generator.

Single emulsion water-in-oil droplets were generated via a standard flow focussing regime, with HFE-7500 + 2% Pico-Surf™ 1 surfactant in the continuous phase. Flow of both phases was adjusted using a LabVIEW controlled Fluigent™ MFCS-100 pressure pump.

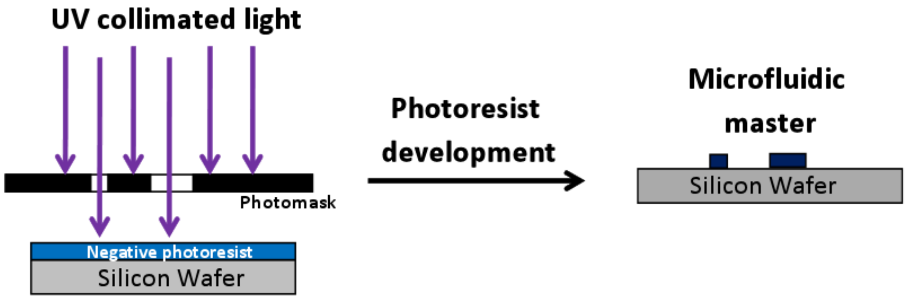

Figure S1: UV Lithography of Negative Photoresist on Silicon Wafer. A 100µm layer of TMMR S2000 photoresist is spin-coated onto a silicon wafer, exposed to collimated UV light, and treated with developer solution to remove non-crosslinked photoresist.

2 Microfluidic Operations

The platform for automated, recursive evolution of microfluidic droplets is a modular system, made up of several devices. In total, there are six independent modules, each of which are fabricated on separate chips. This allows modules to be recombined and connected in a "plug-and-play" manner, affording greater operational flexibility to the system. Two workflows have been established for recursive evolution of micro-droplets. In one workflow, droplets are incubated and then pumped through a passive size sorter (Figure S2.3.1), which selectively distributes droplets to different outlets depending on size, thus isolating several sub-populations of droplets, each of which has a different average fitness. In another workflow, droplets are actively sorted at the individual droplet level using real-time LabVIEW image analysis and air-actuated PDMS valves.

2.1 Droplet Generation

A double droplet generator module allows for simultaneous generation of two micro-droplet populations on the same chip (Figure S2.1.1), which can then be co-incubated on-chip or off-chip in incubation reservoirs, before transfer to other modules (Figure S2.2.1). Droplets were generated using two separate aqueous inputs, the flow rates of which can be adjusted in real-

time using MAESFLO controlled LabVIEW automation. Using this technique, monodisperse micro-droplets can be produced at a high frequency. Integration of a high-speed Mikrotron^{TM} MC1363 camera also enables the ratio of two droplet populations being produced to be monitored.

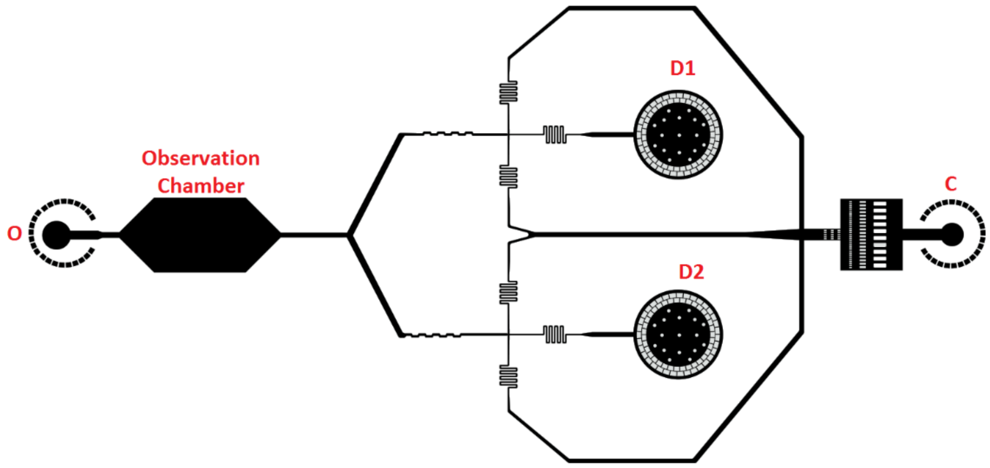

Figure S2.1.1: Microfluidic Droplet Generator Duplex. Two populations of aqueous, single emulsion micro-droplets were generated simultaneously in the same device. Aqueous, disperse phase fluid is injected at two separate inlets (D1 and D2), each of which leads to a flow focussing junction, where the fluid is sheared into droplets by two symmetrically opposing streams of continuous phase (HFE-7500), pumped in through inlet C. Both droplet populations can be observed in an on-chip co-incubation chamber, or transferred directly to other modules via outlet O.

2.2 Droplet Incubation and Transfer

Transfer of droplets between modules is made possible using off-chip incubation reservoirs connected by 1 / 32” PTFE tubing, with air-driven static pressure provided by Fluigent^{TM} pressure pumps. Reservoirs were constructed by punching 7 mm × 10 mm holes in a block of PDMS, which are then plasma bonded to a glass substrate. Thin, 7 mm circles of PDMS were used as covers for the reservoirs.

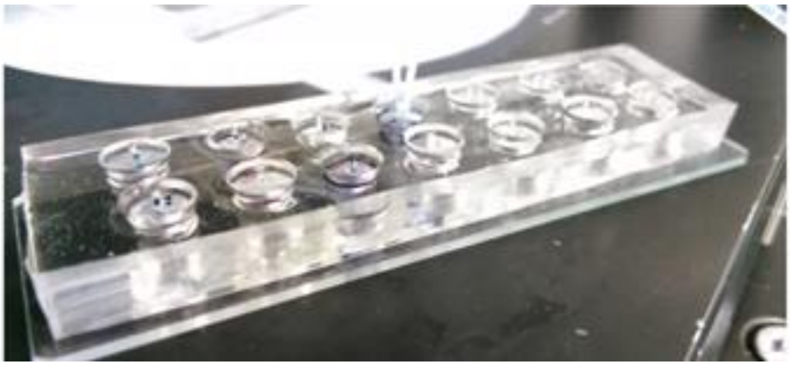

Figure S2.2.1: Off-Chip Droplet Incubation Reservoirs. Home-made inlet-outlet device fabricated by bonding two parts of PDMS and punching two 0.75 mm holes. 1/32’’ OD PTFE tubing was used to connect the reservoir to the microfluidic chip.

2.3 Droplet Size Sorting

Pinched flow fractionation (PFF) is used as a means of passively sorting droplets into sub-populations by size (Figure S2.3.1). The principles behind this technique have been reported by Yamada et al [1]. Droplets can be injected into these size sorting devices directly from incubation reservoirs.

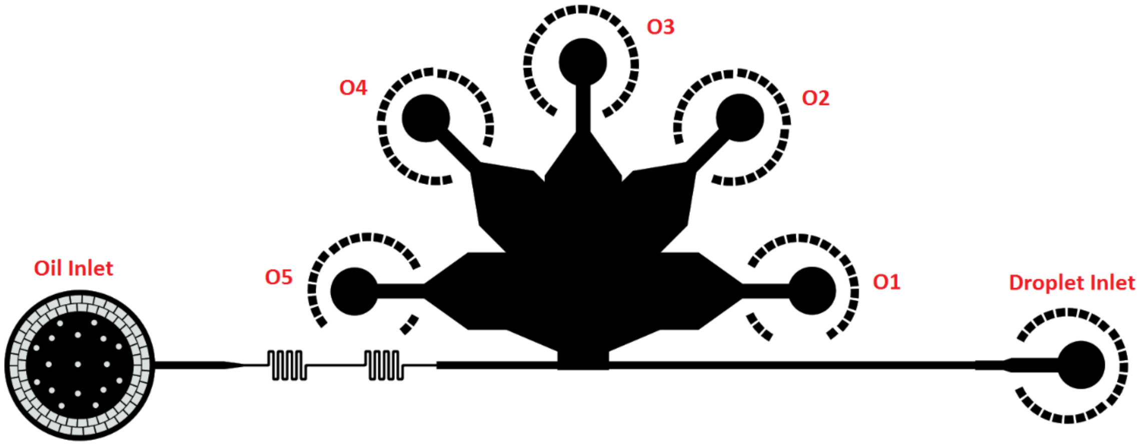

Figure S2.3.1: Pinched Flow Fractionation (PFF) Size Sorting Device. The PFF device has one inlet for injection of a polydisperse micro-droplet population, an inlet for the oil focussing stream, and five outlets in the sorting chamber. The principles of PFF have been described by Yamada et al. [1s].

For active sorting, real-time image processing will be used to screen individual droplets as they pass through a microfluidic channel. If the droplets exceed a pre-defined size threshold for fitness, an air-actuated PDMS valve is activated by input of pressurised air. This causes a constriction in one branch of a bifurcated channel, diverting droplets into the appropriate branch (Figures S2.3.2, S2.3.3). Thin, high-aspect ratio PDMS channels are used to produce the air-actuated valves. High-aspect ratio channels are required to produce valves with sufficient flexibility to constrict neighbouring channels upon flowing of pressurised air.

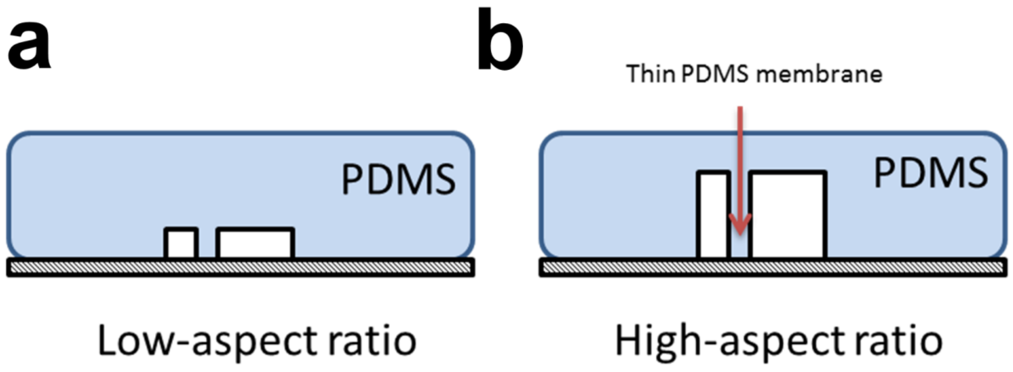

Figure S2.3.2: Microfluidic Device with (a) low-aspect ratio and (b) high-aspect ratio PDMS channels.

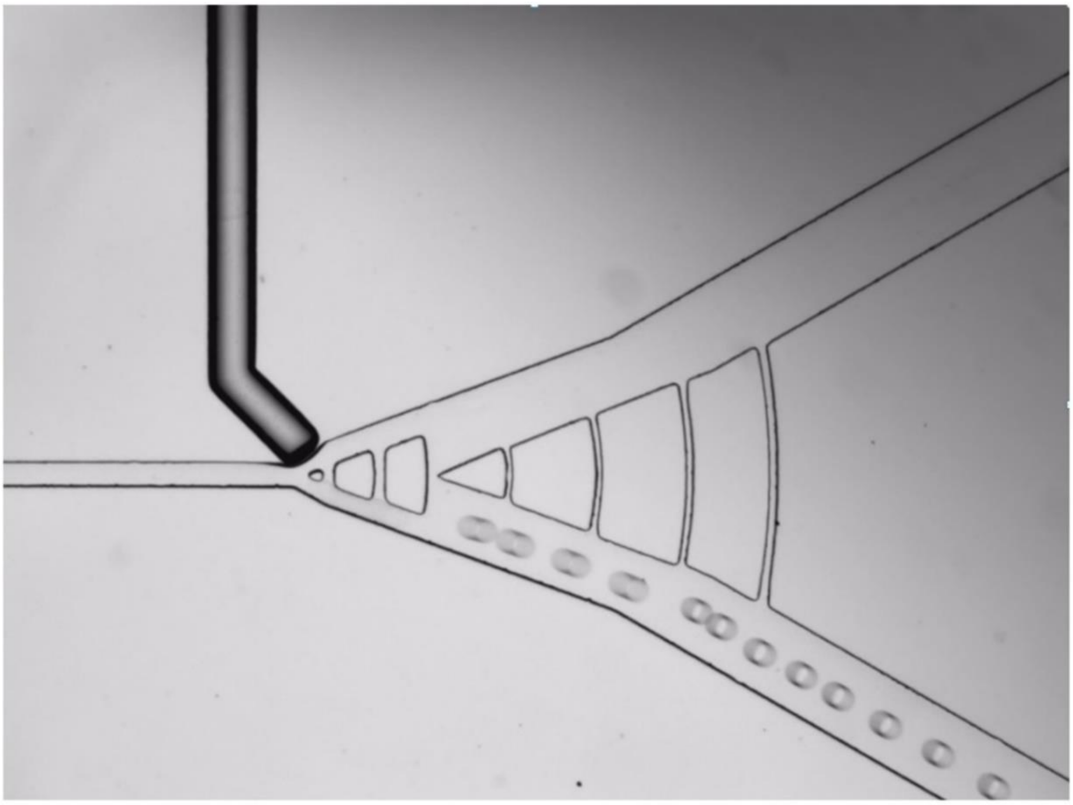

Figure S2.3.3: Active Droplet Sorter. Flowing pressurised air through a high-aspect ratio PDMS channel constricts one branch of a bifurcating channel. This causes droplets to flow selectively into the unrestricted channel.

2.4 Droplet Splitting and Fusing

An integrated droplet splitter and fuser module (Figure S2.4.1) is used to provide droplets that have been sorted via the active size sorter with fresh feedstocks, and divide each into two daughter droplets for re-circulation in the next generation. The droplet fuser component fuses the selected droplets with new droplets containing fresh starting material. Fusion is induced by two microfabricated electrodes, which polarise the droplets and induce coalescence. Electrodes are fabricated by flowing low melting temperature solder through thin PDMS channels, which are connected to a current source via copper wiring.

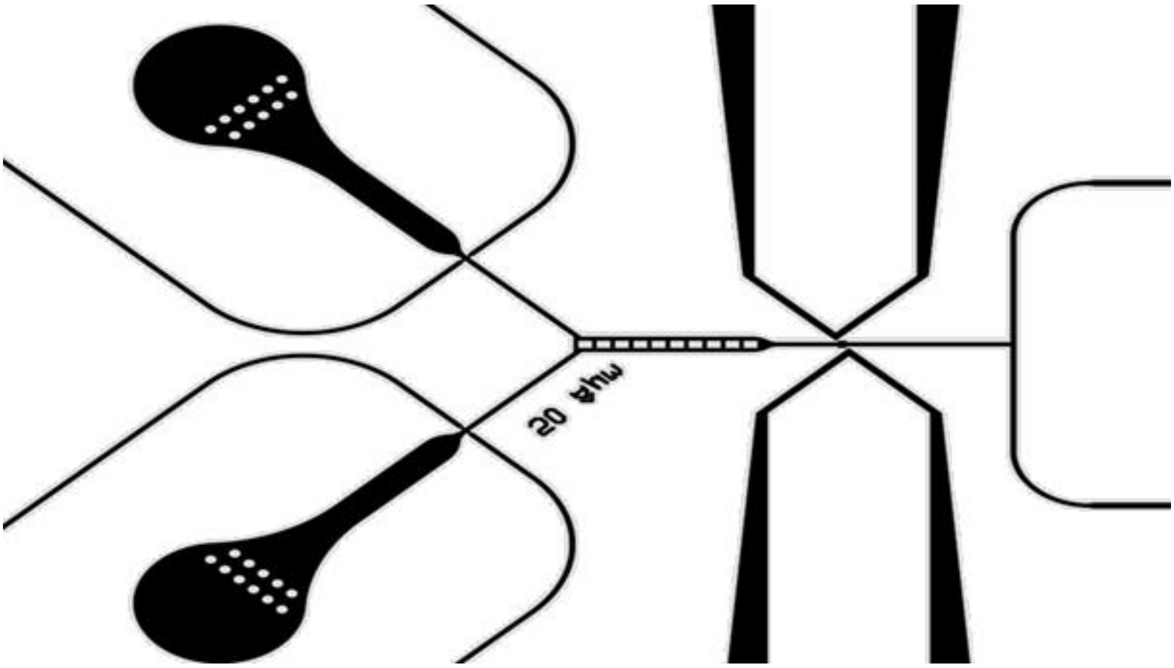

Figure S2.4.1: Droplet Fuser and Splitter. Two inlets allow for reinjection of droplets, which flow through a rail-road synchroniser channel, before being fused by two electrodes and split in a bifurcating channel.

2.5 Osmotic Exchange between Micro-droplets

The basis for evolutionary competition between co-incubating micro-droplets is exchange of reagents between neighbouring droplets and the selective retention of reagents by the fittest droplets (see Figure 3, main text). When this occurs, the fittest droplets exhibit higher osmolarity relative to their neighbours, and thus increase in size due to osmotic exchange (Figure 4, main text). Using LabVIEW image analysis, osmotic exchange can be tracked in real time by measuring average droplet size and polydispersity (Figure 5, main text). A mixed but monodisperse population of 50mM glycylglycine droplets incubating with pure water droplets was used as a model for this process. The glycylglycine droplets exhibited a greater osmotic pressure than the water droplets, and therefore increased in size. This effect was not observed for unmixed droplet populations containing only glycylglycine or pure water.

3 LabVIEW Integration and Image Analysis

LabVIEW was used to control the flow rate of inputs into the various modules in the platform, and to carry out image analysis. Using LabVIEW software integrated with Fluigent™ MAESFLO, multiple air-driven Fluigent™ pumps could be actuated simultaneously and in real time (Figure S3.1). This, combined with LabVIEW-controlled Tricontinental pumps, enabled full automation of the various fluid handling operations required for the platform.

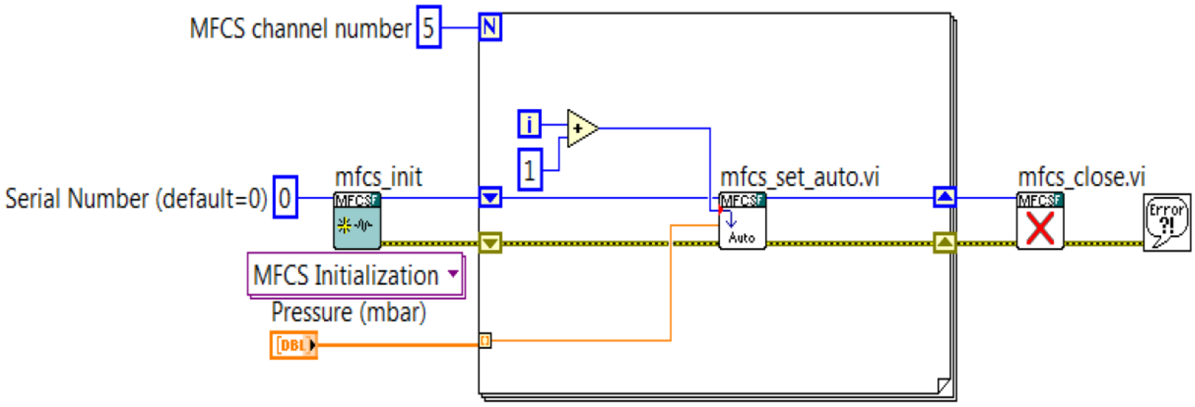

Figure S3.1: LabVIEW Controlled Fluigent Actuation. An example visual instrument (VI) in which the pressure of several Fluigent^{TM} pumps are controlled simultaneously and in real time.

Image acquisition and analysis was also fully integrated into the automated system. Live images were recorded using a Mikrotron^{TM} MC1363 high-speed camera, and analysis of droplet size and polydispersity was carried out using a circle detection algorithm on LabVIEW Vision Assistant. In the fully automated image analysis process, circles were overlaid onto the boundaries of micro-droplets (Figure S3.2, Figure S3.3). The average diameter and polydispersity of micro-droplet populations was inferred from the dimensions of the overlaid circles.

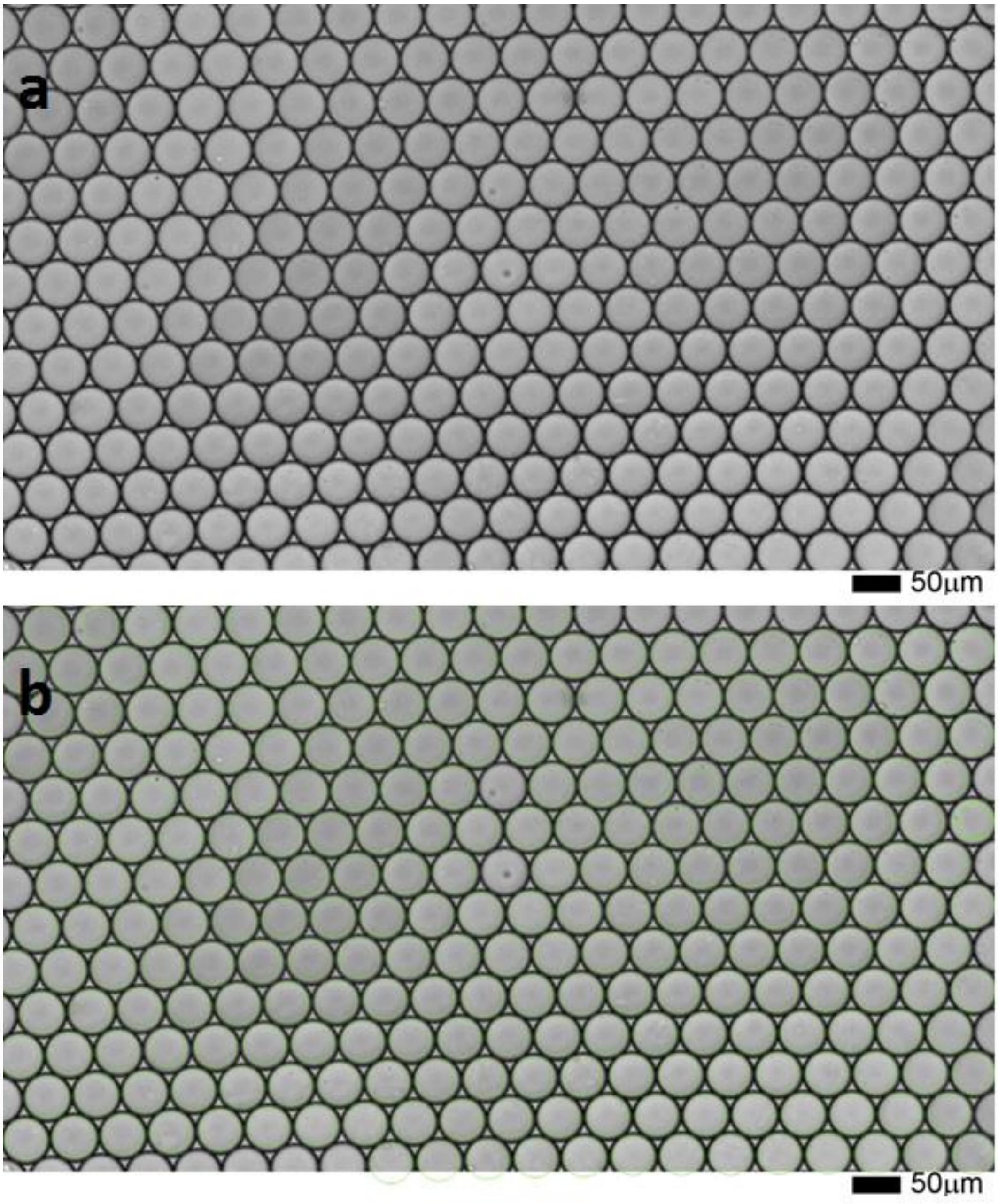

Figure S3.2: Boundary Overlay of Micro-droplets.

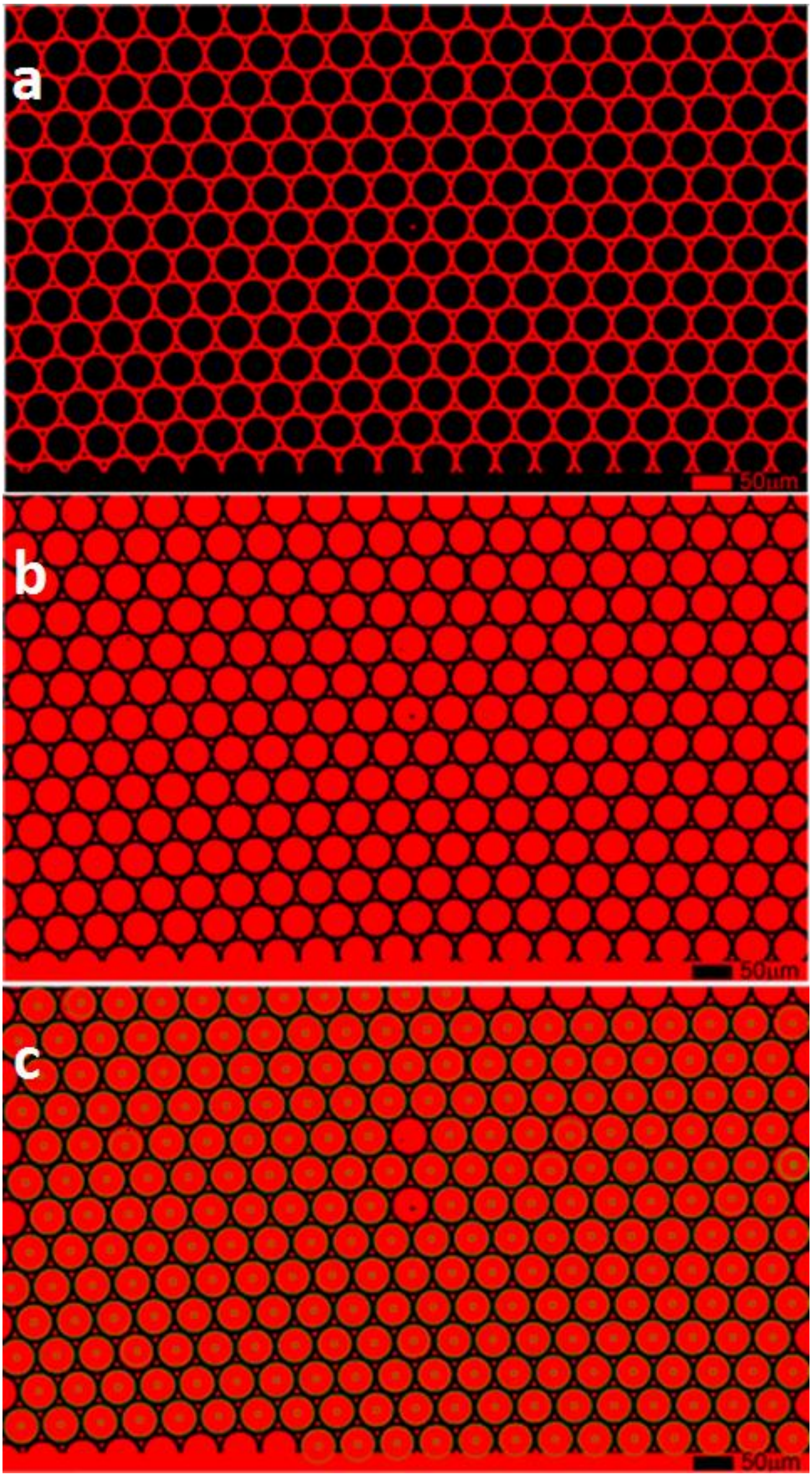

Figure S3.3: LabVIEW Vision Assistant Image Processing for Circle Overlay. Images were colour thresholded (a), inverted (b), and run through a circle detection algorithm (c).

4 Instrumentation and Materials

Spin coater (Laurell, model WS-650MZ-23NPP).

Oxygen plasma cleaner (Harrick-Plasma, Model PDC-001).

UV light source (NewPort, Flood Exposure source, 500 W Hg, model 97435)

Silicon wafers (100 diameter, p-type, 1S polished; Pi-Kem).

Glass slides (TAAB)

1/32'' OD PTFE microtubing (Cole Parmer, PTFE#30)

Biopsy punches (0.75 and 7.0 mm diameter, Harris Uni-Core)

SU-8 developer (MicroChem).

PDMS (Sylgard 184, Dow Corning).

HFE-7500 Oil (3M)

Trichloro (1H,1H,2H,2H-perfluorooctyl) silane (Sigma)

TMMR^{TM} S2000 photoresist (Tok)

Pico surf 1 surfactant (Dolomite Microfluidics)

Low-melting temperature solder: Indalloy 78 Bismuth-Lead-Tin Solder Alloy (Indium Corp.)

Bright-field inverted microscope (Olympus)

LabVIEW software (LabVIEW Core and LabVIEW field-programmable gate array (FPGA); National Instruments)

5 References

1. Yamada, M., Nakashima, M. & Seki, M. *Anal. Chem.* **2004**, **76,** 5465–5471. doi: 10.1021/ac049863r.
